# More Than Just Privacy: Using Contextual Integrity to Evaluate the Long-Term Risks from COVID-19 Surveillance Technologies

**DOI:** 10.1177/2056305120948250

**Published:** 2020-07-30

**Authors:** Jessica Vitak, Michael Zimmer

**Affiliations:** 1 University of Maryland, USA; 2 Marquette University, USA

**Keywords:** privacy, contextual integrity, COVID-19, contact tracing, surveillance

## Abstract

The global coronavirus pandemic has raised important questions regarding how to balance public health concerns with privacy protections for individual citizens. In this essay, we evaluate contact tracing apps, which have been offered as a technological solution to minimize the spread of COVID-19. We argue that apps such as those built on Google and Apple’s “exposure notification system” should be evaluated in terms of the contextual integrity of information flows; in other words, the appropriateness of sharing health and location data will be contextually dependent on factors such as who will have access to data, as well as the transmission principles underlying data transfer. We also consider the role of prevailing social and political values in this assessment, including the large-scale social benefits that can be obtained through such information sharing. However, caution should be taken in violating contextual integrity, even in the case of a pandemic, because it risks a long-term loss of autonomy and growing function creep for surveillance and monitoring technologies.

## Introduction

Since 2016, we have been collaborating to understand how people make sense of and respond to privacy risks of new technologies. We focus our evaluations on mobile technologies because they are both ubiquitous and raise unique privacy challenges. Smartphones, wearables, and Internet of things (IoT) technologies are designed to make people’s lives easier, more efficient, and healthier. To do this, they collect a wide range of data, including location and movement data, health and fitness data, and social media content. Data typically flow constantly from our devices to outside parties, and most consumers have little understanding of what data are being shared, who gains access to that data, and what they use that data for.

When considering surveillance and monitoring in response to COVID-19, we recognize that, in times of crisis, the norms around acceptable data flows may shift. At the same time, there is a risk that temporary measures established during a crisis become permanent and unnecessarily reduce citizens’ privacy, which was the case in the United States following the September 11 terrorist attack. For a public health crisis like COVID-19, proposed solutions to minimize the spread of the virus include a number of potentially invasive forms of data collection.

In this essay, we examine how contact tracing apps are being developed and rolled out. We identify privacy concerns about apps being framed as solutions to reduce the risk of COVID-19 and enable the reopening of communities and businesses. Using Nissenbaum’s (2010) framework of privacy as contextual integrity (CI), we argue that the appropriateness of sharing data with third parties to support public health will be contextually dependent. Furthermore, deciding when to violate information norms that determine appropriate information flow of health data should also be governed by prevailing social and political values. If we ignore this second point, we risk a long-term loss of autonomy and growing function creep across a wide range of technologies.

## Contact Tracing and Privacy Concerns

Contact tracing refers to the practice of mapping out who an infected person has been in contact with to minimize disease spread. It has been used in prior disease outbreaks, including Ebola in 2014 (Webb et al., 2015) and the HIV/AIDS epidemic (Adler & Johnson, 1988). Contact tracing involves three steps: (1) identifying people who had been in contact with an infected person, (2) locating and notifying contacts about their exposure, and (3) regularly following up with contacts to monitor for infection (World Health Organization, 2017). Contact tracing has traditionally been conducted using large staffs of workers to canvas neighborhoods on foot; however, newer technologies should make this process faster and more efficient.

In the weeks following the declaration of COVID-19 as a pandemic by the World Health Organization, several companies and countries have unveiled plans for smartphone-based contact tracing apps. Most notably, Google and Apple—who control the two most popular smartphone operating systems—announced a collaboration to develop tools to fight the virus (Romm et al., 2020). Their “exposure notification system” API uses Bluetooth (rather than more precise location data) to identify other smartphones that come into proximity with the phone of an infected person, then notify them through the app. Similar solutions using Bluetooth are being used in other countries, including the United Kingdom (Harkness, 2020), as well as Singapore and Australia (Abbas & Michael, 2020).

Google/Apple’s proposed tool has been largely received with support from privacy scholars regarding the steps being taken to ensure data privacy and security (Whittaker, 2020). That said, there are a number of challenges to balancing citizen privacy with public health benefits. First, while Bluetooth is a more privacy-preserving choice, it is also less useful at detecting potential virus exposure. False positives may be very common, depending on factors like whether a phone is in someone’s pocket or whether people are outside versus inside (O’Neill, 2020). Beyond that, as many as 2 billion older mobile phones will not be able to use contact tracing apps, and those left out include some of the most vulnerable populations (Bradshaw, 2020).

The biggest privacy concern with contact tracing, however, is the appropriateness of data flows from users’ smartphones: who can access data, how long is data stored, and for what purposes could that data be used in the future. Numerous groups have pointed to the widespread surveillance policies enacted in the United States following the September 11 terrorist attacks as a cautionary tale for the normalization of mass surveillance, and have argued for careful assessments of current surveillance tools to protect citizens’ civil liberties (e.g., Díaz, 2020; Friedersdorf, 2020).

## Ensuring Appropriate Data Flows in Contact Tracing

The theory of CI (Nissenbaum, 2004, 2010) rejects the traditional dichotomy of public versus private information, as well as the notion that privacy preferences and decisions in one setting universally apply to other settings. Instead, CI rests on the understanding that our interactions—with other people, institutions, and technologies—occur in particular contexts, and norms of appropriateness govern people’s expectations of how personal information should flow within a given context.

We have seen this in our research on people’s privacy attitudes regarding their personal fitness trackers (Zimmer et al., 2020). Our interviewees—recruited from a random sample of staff at two American public universities—had distinct privacy expectations for different types of data generated by their Fitbit, seeing data like steps as more acceptable for the device to collect and share than more personal identifiers. Furthermore, while our interviewees were comfortable with data being shared with Fitbit or their health provider, they felt sharing data with employers or insurance companies was inappropriate.

These expectations about information flows fit with the CI framework, which requires the assessment of four key parameters that shape norms of appropriateness: context, actor, attributes, and transmission principles. *Context* is the backdrop that informs which set of norms govern an interaction. Doctor–patient conversations often occur in a health context while supervisor–employee conversations occur in a workplace context. *Actor* refers to the parties involved in a given interaction. Using our above example, Fitbit sends data about an individual’s step count and heart rate to various recipients, including the user and company servers. *Attributes* are the different types of information in play. Fitbits collect several attributes, including steps, heart rate, and sleep data. Finally, *transmission principles* shape or constrain the flow of information. The principle of confidentiality surrounds doctor–patient conversations, and the principle of consent often applies to a company’s use of an individual’s data.

When a new technology or practice affects these parameters in a way that shifts informational norms, CI may be violated, pointing to a potential privacy violation. Thinking about privacy through the lens of CI forces us to reject simplistic arguments like “since you’re sharing health information with some, you’re okay with sharing it with anyone.” Rather, if one’s personal fitness data were suddenly shared with their employer, the change in *actor* violates the CI of the appropriate information flow. Similarly, a sudden change in the terms of service might disrupt the *transmission principles* previously in place. Or, if a software update now allows the inference of sexual activity based on your fitness metrics, that change in *attributes* means previous appropriateness of information flows from the device to one’s social network might now be disrupted. CI, then, provides us a much more nuanced insight into the appropriateness of changes to personal information flows.

So, what does this mean in terms of privacy concerns with contact tracing and COVID-19? CI helps us explain the strong negative reaction to initial media reports of governments seeking to track people’s locations via their smartphones (Newton, 2020; Tau, 2020). While individuals are comfortable sharing their location with Google or Garmin to receive services like navigation and fitness tracking, having that data flow to government officials for long-term monitoring of citizen movements—a change in *actor* and *transmission principles*—was deemed an inappropriate new information flow.

CI also explains why the solution proposed by Google/Apple *has* been largely considered acceptable. By relying on Bluetooth to track physical proximity between phones, the system does not collect or store any locational data, nor does it send data to centralized databases. Google/Apple have resisted pressure from governments who want access to app data to build a picture of population movements in aggregate. Thus, Google/Apple have created *transmission principles* aligned with users’ expectations, limited the *attributes* of data being used to trace contacts, and restricted *actors* who get access to such information. In all, their solution maintains the CI of appropriate information flows, even when faced with the pressures of managing a public health crisis.

## Protecting Broader Moral and Political Values

Even with CI being preserved through Google/Apple’s approach, nagging concerns remain about the broader privacy and surveillance implications of embracing *any* platform intended to monitor people’s movements and health status. As Díaz (2020) wrote for the Brennan Center:

The impulse to turn to high-tech tools in this time of crisis is understandable—and some such tools might indeed be a useful part of our response to Covid-19. At the same time, history offers ample reason to proceed with caution.

Díaz notes how many post-9/11 surveillance programs are still active today, nearly two decades after the crisis that justified their creation. Faced with a history of technology providers and governments alike misusing personal information, extending data collection beyond what was initially authorized or envisioned, and engaging in “function creep”—when a technology deployed for benign purposes slowly gets repurposed for problematic ends—it is reasonable to be concerned that we are opening pandora’s box by embracing any smartphone-based contact tracing solution.

CI helps us work through these broader concerns about future data misuse. Our assessment of the key parameters shaping norms of appropriate information flows is only the starting point for determining whether CI may be disrupted by new technology. This initial analysis provides us a prima facie judgment as to whether a new process significantly violates the entrenched norms within the context.

Nissenbaum (2010) also argues that CI demands a wider examination of the *moral and political implications* of new information flows to make a more complete assessment as to whether technology should be allowed or resisted. In her words, to properly apply CI, we must

consider moral and political factors affected by the practice in question. What might be the harms, the threats to autonomy and freedom? What might be the effects on power structures, implications for justice, fairness, equality, social hierarchy, democracy, and so on? (p. 182)

Clearly, we are attempting to protect public health amid a global pandemic, and we are balancing that goal against individual privacy. Our *prima facie* assessment of the Google/Apple solution suggests sufficient privacy protections are in place. But there are also broader moral and political values at play when thinking about appropriate data flows, including individual autonomy and freedom from surveillance. We must ask, What are the prevailing values, goals, and ends within the context of using smartphones for contact tracing? CI pushes us to weigh these values when making the final determination of whether the Google/Apple contact tracing is acceptable.

Google/Apple have taken careful steps to protect individual privacy; our fear, however, is that despite their best efforts, embracing such forms of widespread surveillance to address the pandemic might be used to justify wider escalations of health monitoring that impinge on individual autonomy. Not all tracing apps will be as privacy-protecting as the Google/Apple solution, and other technologies might collect more detailed health data, track more specific locational data, or rely on face recognition to identify and monitor infected citizens. Using CI, we must look beyond near-term privacy-preserving steps taken by Google/Apple, and seriously reflect on possible future impacts on broader moral and political values as these technologies proliferate.

We encourage technology developers and policymakers to embrace a set of principles to ensure CI is maintained to the fullest extent. These include implementing use limitations to avoid function creep, having strong data minimization and destruction policies, and ensuring full transparency and accountability. Such policies, at a minimum, must be in place to ensure that any contact tracing app will accord with protecting the broader set of moral and political values that CI compels us to protect.
